# International remote collaboration enabled inaugural endoscopic sleeve gastroplasty in Japan

**DOI:** 10.1002/deo2.31

**Published:** 2021-08-25

**Authors:** Akira Dobashi, Kohei Uno, Hiroaki Matsui, Hiroto Furuhashi, Toshiki Futakuchi, Shunsuke Kamba, Shingo Ono, Naoto Tamai, Atsushi Watanabe, Christopher J. Gostout, Kazuki Sumiyama

**Affiliations:** ^1^ Department of Endoscopy The Jikei University School of Medicine Tokyo Japan; ^2^ Department of Surgery The Jikei University School of Medicine Tokyo Japan; ^3^ Developmental Endoscopy Unit, Division of Gastroenterology and Hepatology Mayo Clinic Rochester Minnesota USA

**Keywords:** endoscopic sleeve gastroplasty, remote, obesity, Japanese

## Abstract

Obesity causes multiple conditions such as type 2 diabetes, cardiovascular disease, and so on, and an intervention is needed for controlling weight and improving metabolic syndrome. However, the effectiveness of lifestyle interventions and pharmacotherapy are restrictive for losing weight. Endoscopic sleeve gastroplasty (ESG) was developed as a new therapy, picking the best of both medication and surgery, less invasive and more effective. Recently, ESG is gradually spreading in Western countries, but there is Case report doesn't need conclusion/result for Japanese patients. We herein reported the first clinical case of ESG in Japan.

Given the situation of the pandemic of COVID‐19, we could not invite a proctor from Western countries and receive the instruction of the device setting and maneuver face to face. Thus, we conducted the training for device setting, maneuver, and operation under a web‐based international remote collaboration. Eventually, we completed ESG without an adverse event. We could prove this web‐based proctor system was useful through the introduction of ESG in Japan. The international remote collaboration could become a new normal even in the endoscopy field post‐COVID‐19 era.

## INTRODUCTION

Obesity is associated with co‐morbidities such as type 2 diabetes, cardiovascular disease, nonalcoholic steatohepatitis, cerebral stroke, and increased risk of cancer.^1^ Gastrointestinal weight loss surgery (GIWLS) such as Roux‐en‐Y gastric bypass and laparoscopic sleeve gastrectomy (LSG) has been developed for whom lifestyle interventions and pharmacotherapy are ineffective. Widespread application has been limited to less than 2% of the eligible population due to costs, morbidity, and mortality associated with GIWLS procedures.^2^

Endoscopic sleeve gastroplasty (ESG) was reported in 2013.^3^ This peroral flexible endoscopic procedure avoids the need for skin incision and permanent alteration of the upper GI tract notably with significantly fewer serious adverse events.^4^ Recently long‐term outcomes were reported, with mean total body weight loss maintained at 15.9% over 5 years.^5^ ESG has positioned itself in Western countries as a bridge between highly invasive surgical and standard medical treatment. ESG has just started in East Asia. Our plan is to introduce ESG in Japan through a clinical trial examining safety and efficacy. We planned to preliminarily conduct supervised ex vivo and in vivo animal training with the requisite endoscopic suturing system (OverStitch Sx; Apollo Endosurgery, TX, USA) to master full‐thickness suturing and the ESG technique. We planned for ESG to be done in the presence of an expert proctor.

At the beginning of 2020, the coronavirus disease 2019 (COVID‐19) became a worldwide pandemic^6^ and eliminated any opportunity to meet in the face, especially with international colleagues. Thus, we introduced this new therapy with remote‐based training and proctoring. Herein, we present the first ESG case in Japan using international remote collaboration. This case experience may provide valuable insight into remote‐based collaboration for introducing new endoscopic therapy in a new normal world post‐COVID‐19.

## CASE PRESENTATION

The first ESG case in Japan was performed within a clinical trial examining the efficacy and the safety of ESG for Japanese obese patients (jRCTs032200049). This study was approved by the Certified Review Board of the Jikei University School of Medicine and was compliant with the Declaration of Helsinki. Written informed consent was obtained from the participant. The patient was a 34‐year‐old Japanese male. In addition to obesity, his medical history included malignant hypertension, type 2 diabetes, hyperlipidemia, and fatty liver. The BMI was 34.4 kg/m^2^, and he was classified as obesity grade I. He underwent lifestyle intervention and pharmacotherapy at Jikei University Hospital without success. Then, he selected ESG. Screening blood antibody test and endoscopic examination showed no active or history of *Helicobacter pylori* infection.

## INTERNATIONAL REMOTE COLLABORATION FOR ANIMAL‐BASED DEVICE TRAINING AND ESG PROCEDURE

OverStitch Sx is the most recent version of an endoscopic suturing system allowing full‐thickness suturing critical for ESG.^7^ However, the device had never been used in Japan. This device was chosen because it could be mounted on a variety of single‐channel endoscopes. The setup and performance of this device differed compared to our earlier experience with the OverStitch Gen 2 device requiring a dual‐channel therapeutic Olympus endoscope.^3^ Initially, we planned to invite a western endoscopist with expertise in ESG and learn the newer device use and nuances. The first clinical case was to be done under real‐time supervision by the same proctor. However, these plans were thwarted by the COVID‐19 pandemic. We then decided on remote instruction and clinical proctoring. GoToMeeting (LogMeIn, Inc. Boston, MA, USA) and Zoom (Zoom Video Communications, Inc, San Jose, CA, USA) provided remote collaboration. Video imaging transmitted included endoscopic imaging, endoscopist physical actions, and procedure room activity. Staff from Apollo Endosurgery joined in the ex vivo training course from France, UK, and Italy.

## PRE‐CLINICAL TRAINING

**FIGURE 1:**
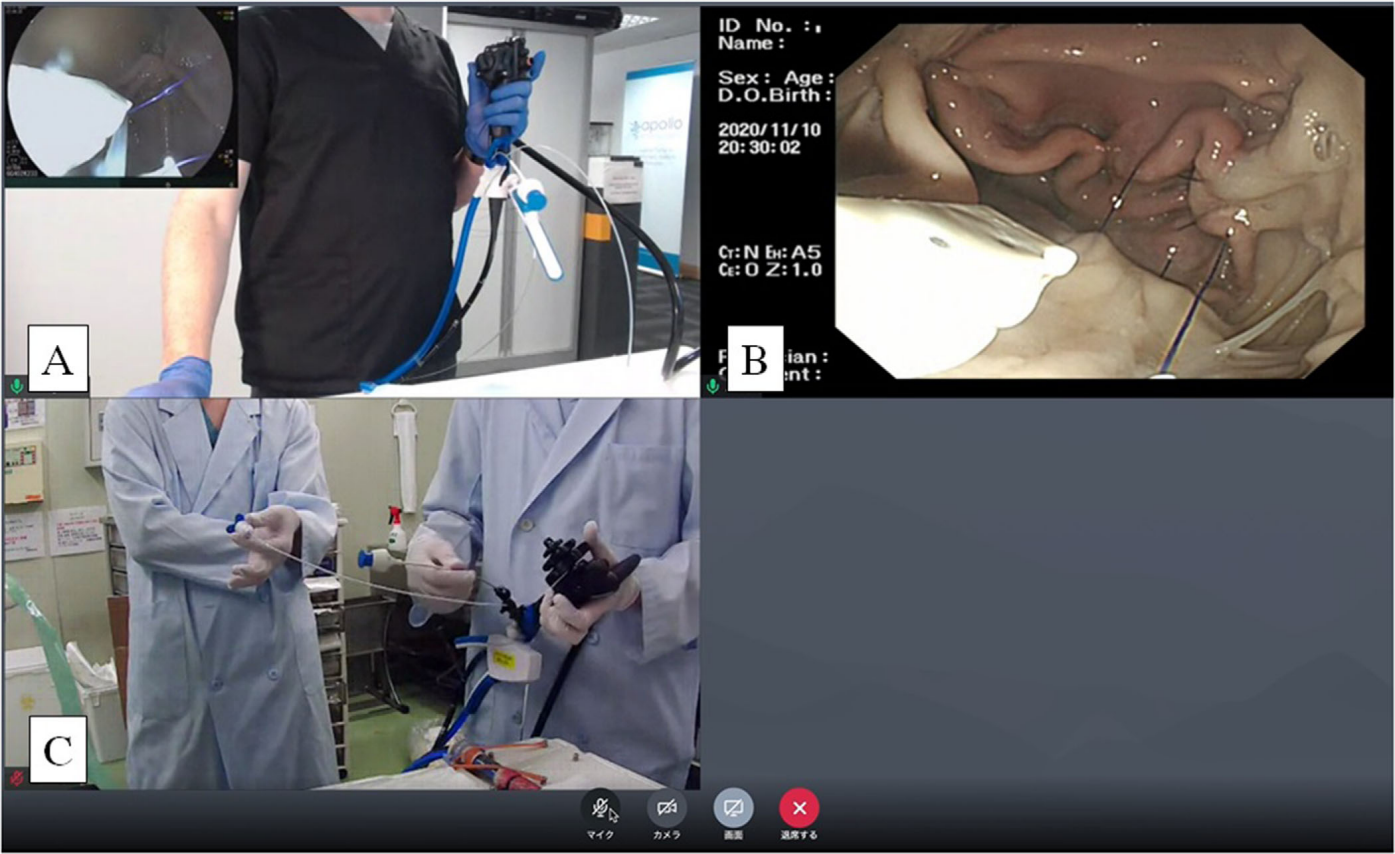
Ex vivo training session using a resected porcine stomach via internet collaboration between Japan and European countries. (a) Endoscopic image and hand positioning view from an instructor in the UK. (b) An Endoscopic image of a trainee in Japan. (c) Hand view of the trainee in Japan

Three half‐day sessions focused on device setup and operation. Day 1 involved set‐up, basic device operation, and takedown.^7^ Day 2 involved the use of an ex vivo porcine stomach mounted in an endoscopy upper GI tract training tray (Endo‐X‐Trainer; Medical Innovations Inc., Rochester, MN, USA) with an esophageal overtube (20 double slim 16406; TOP Corporation, Tokyo, Japan) to acquire predictable placement of full‐thickness stitches and creation of the volume reducing plication essential to the ESG (Figure 1). Day 3 involved performing ESG in a live animal. The animal was under general anesthesia with endotracheal intubation along with overtube intubation of the esophagus. An ESG expert (Christopher J. Gostout) monitored all of the video streams described above throughout the session, providing instruction and advice. Two endoscopists used an animal each.

## CLINICAL ESG CASE

**FIGURE 2:**
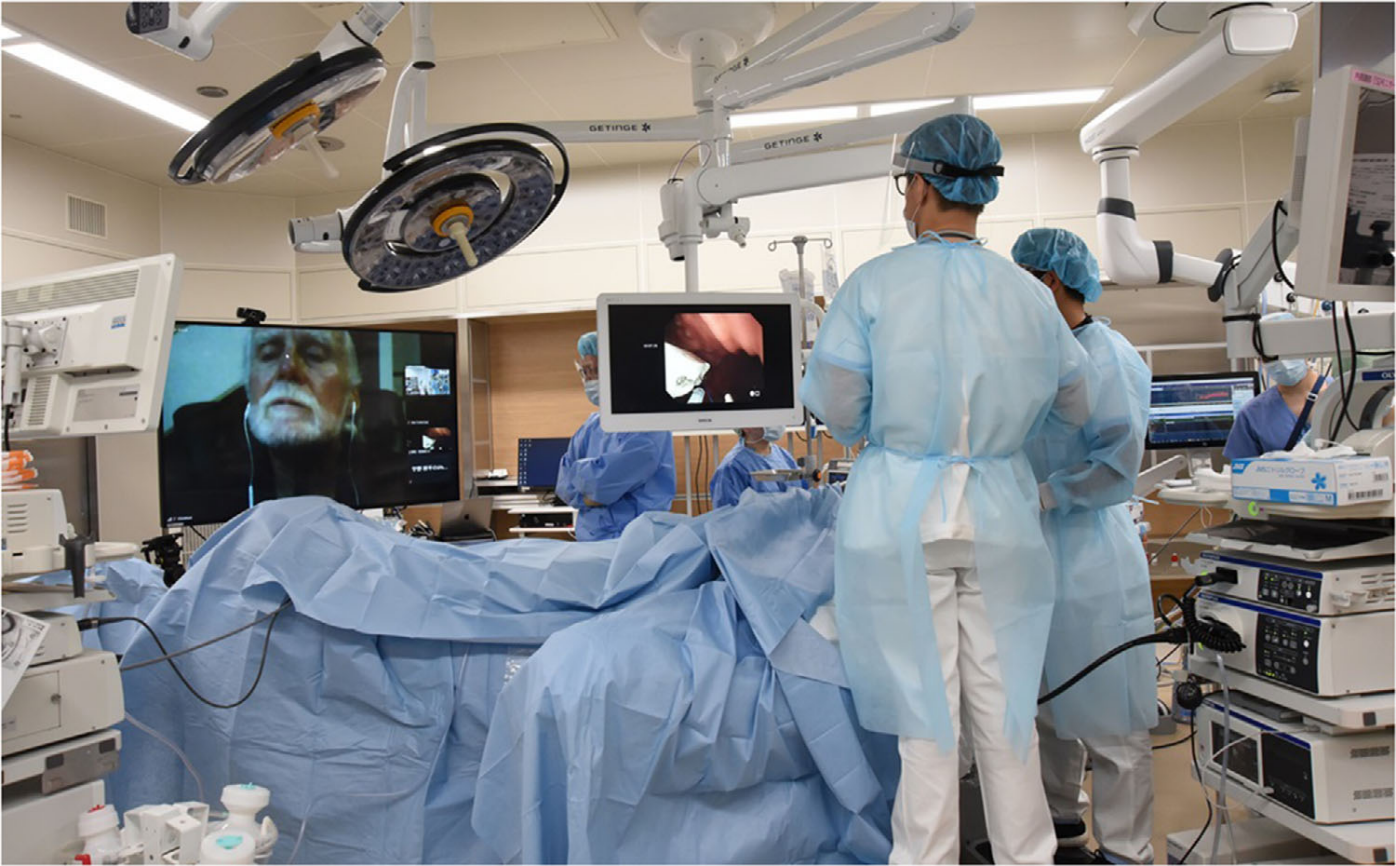
Endoscopic sleeve gastroplasty (ESG) performed under proctor supervision in the United States. The operators and the proctor could share the real‐time endoscopic image, and the operators could receive the instruction while ESG is being performed

**FIGURE 3:**
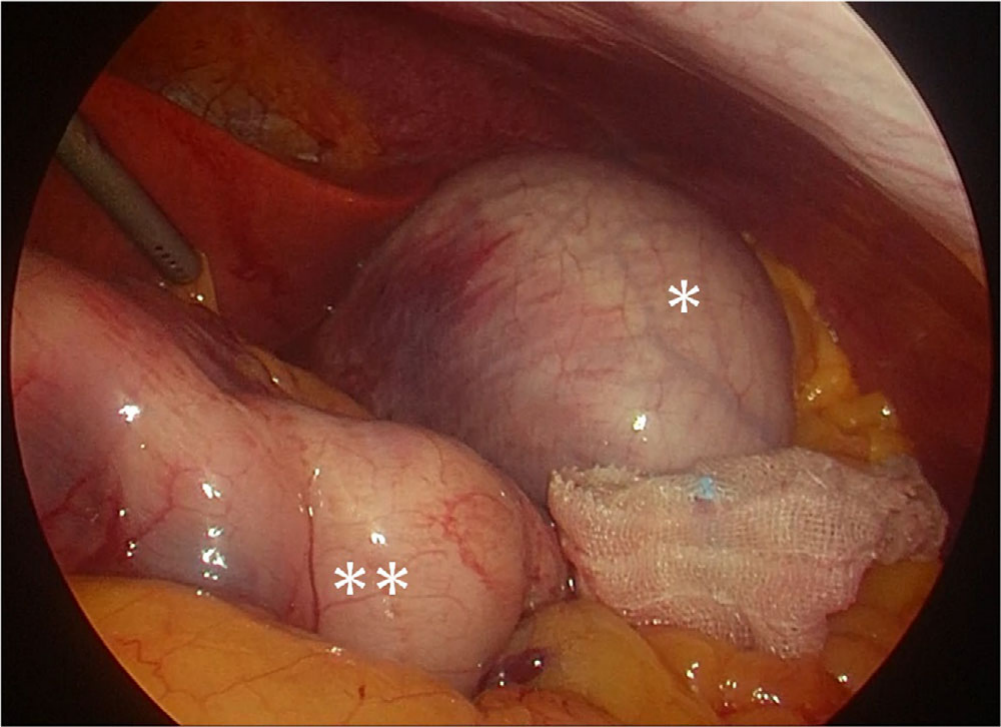
The laparoscopic view after endoscopic sleeve gastroplasty (ESG). There was no other organ injury. The full‐thickness suturing at the body outside of the stomach was observed: *the gastric fundus, **the reduced gastric body

The ESG was performed in an operating room with the virtual expert endoscopist (Christopher J. Gostout) proctoring the entire operation, simultaneously observing the endoscopic image, the endoscopists, and operation room activity using multiple cameras via Zoom (Figure 2). The patient was maintained under general anesthesia with endotracheal intubation. Unique to this case, the patient was prepped for laparoscopy to inspect the stomach and surrounding structures after completion of the ESG. A 20 mm diameter overtube (20 double slim 16406) was inserted into the esophagus. Cautery markings were placed on the anterior and posterior wall by an endoscopic submucosal dissection knife (Dual knife; Olympus, Tokyo, Japan) from the incisura proximally, short of the fundus, to target the placement of the stitches creating each of the individually stacked plications. Plications were created beginning at the incisura and stopping before the fundus. Stitches were placed using a pattern beginning at the mid anterior wall to the greater curvature, then a mid‐posterior wall, back to the greater curvature, and finally the anterior wall.^7^ Additional in‐line stitches were placed approximately 1 cm after the first anterior wall stitch and posterior wall stitch to foreshorten the stomach. Seven stitches were placed for each plication using this suturing pattern. Four plications were created with four sutures. Two additional sutures were placed with three stitches (anterior, greater curve, and posterior) overlying the two proximal plications for reinforcement. The laparoscopy after the ESG observed no organ injury and a reduced stomach (Figure 3).

**FIGURE 4:**
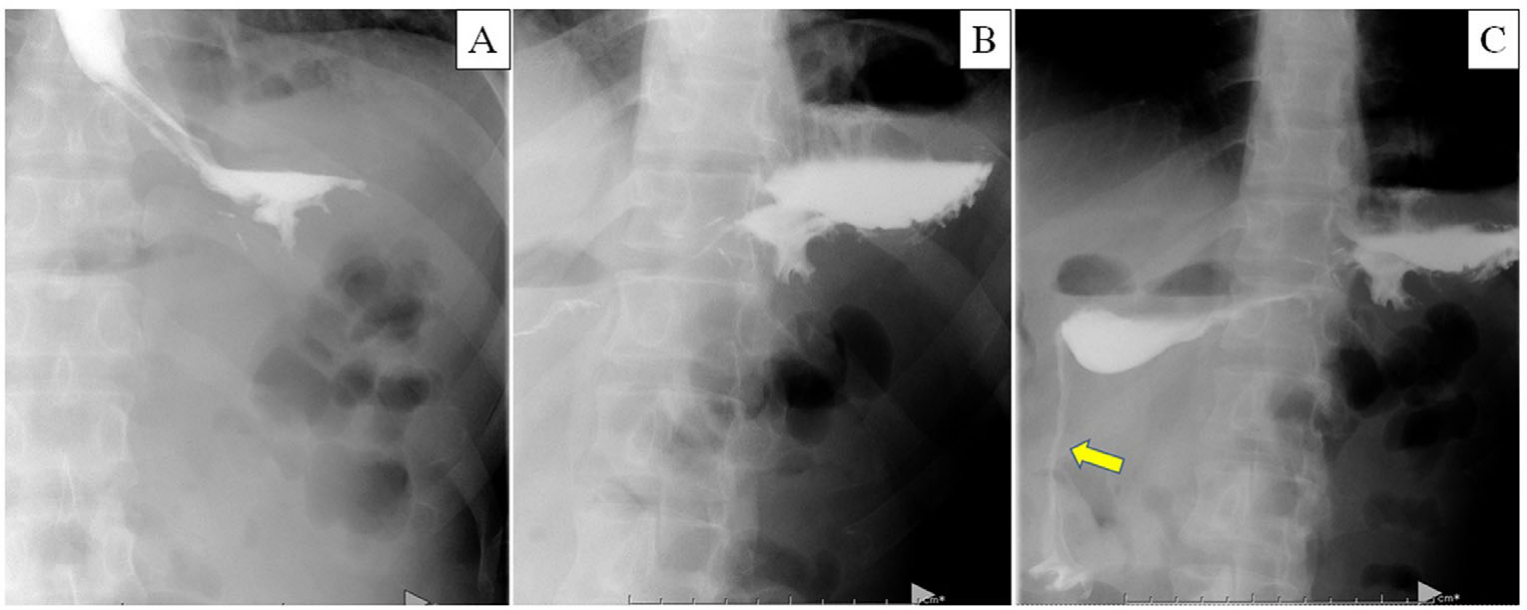
Fluoroscopy on the post‐operation day 1 showed a sleeve‐shaped stomach, and we saw the delayed emission of contrast agent into the duodenum. (a) The time of drinking contrast agent. (b)The stomach showed sleeve‐shape in 2 min. (c) The contrast agent was eliminated into the duodenum in 3 min and flowed into the duodenum (yellow allow)

Post‐operatively, the patient experienced mild epigastric pain, controlled with nonsteroidal anti‐inflammatory drugs (NSAIDs). At postoperative day 1, fluoroscopy of the stomach was done to evaluate any leakage and assess the volume of the stomach (Figure 4). The stomach showed a sleeve‐shaped with delayed emptying. The patient started eating a liquid diet on postoperative day 2 and was discharged on day 3 without adverse events.

## DISCUSSION

This was the first clinical case report of ESG in Japan, safely and effectively performed with an immediate outcome confirmed by laparoscopy. We were unable to have face‐to‐face training and proctoring to introduce this new therapy due to the pandemic. However, we were able to overcome this difficult situation by means of remote international collaboration.

ESG using an endoluminal full‐thickness suturing device was reported by the Mayo Clinic for the first time in 2013,^3^ with many patients subsequently receiving this treatment, especially in the West. On the other hand, there has been no reported experience with ESG from East Asia. This may be due to the low incidence of obesity as reflected by the number of GIWLS in Asia, fewer than in Western countries.^8^ GIWLS is a highly invasive treatment for obesity. LSG is covered by insurance for patients with a BMI of more than 35 kg/m^2^ in Japan. It is difficult to offer GIWLS for obese patients below a BMI of 35 kg/m^2^ due to the extensiveness of the surgery. Asian patients can develop metabolic syndrome when only overweight with a BMI of less than 25 kg/m^2^.^9^ ESG is appealing for these lower BMI patient groups offering greater effectiveness over medication and less invasive than GIWLS with significantly lesser risk for serious adverse events.^4^ Given these considerations, there are many potential patients suitable for ESG in Asian countries. Once the overweight and the obese patient develops metabolic syndrome, there is a commitment to life‐long medication use with surrounding tissues, including compliance. The cost for life‐long pharmacotherapy for single and multiple comorbidities is significant and puts pressure on providing public financing. ESG offers an opportunity to reduce these medical expenses. For these reasons, we advocate including patients in Asia with a BMI of less than 35 kg/m^2^ as candidates for ESG. It is important to acknowledge the value to intervene in these patients before the metabolic syndrome has developed. Accordingly, we targeted this ESG trial for patients with a BMI of more than 32 kg/m^2^.

The difference between the West and Asia is the rate of obesity for the former and the prevalence of gastric cancer for the latter. When ESG is considered for Asian patients, those with a high risk of gastric cancer must be excluded. More than 99% of gastric cancer is related to *H. pylori* infection in Japan.^10^ *Helicobacter pylori* infection status and atrophic gastritis must be assessed in candidates before ESG.

The Web‐based proctor system requires the operator to be fully trained and familiar with the suturing device. This includes recognition of potential problems and understanding the troubleshooting corrections needed, with practice experience for the identified potential problems. The absence of a physically present proctor eliminates the possibility for active intervention should a situation arise benefiting from a “hands‐on” intervention. The restrictions imposed for the proctor in observing the flow of activity within the procedure room, including responding to procedure room personnel questions may have some subtle downsides. The data transmission speed may become another problem. We should make sure the network environment in advance. We used 4G broadband in this case, and we did not feel a delay in the telecommunications between a proctor and trainee.

Given the limitations imposed by the COVID‐19 pandemic, we successfully introduced ESG into Japan recruiting a web‐based international collaboration to overcome the limitations of in‐person traditional training and proctoring for a new procedure along with the absence of prior clinical suturing experience.

## CONFLICT OF INTEREST

Christopher J Gostout is a chief medical officer at Apollo Endosurgery. All other authors have no conflict of interest.

## FUNDING INFORMATION

This clinical trial is being done with a grant from Apollo Endosurgery.

## References

[1] Pi‐Sunyer X , Blackburn G , Brancati FL , *et al*. Reduction in weight and cardiovascular disease risk factors in individuals with type 2 diabetes: One‐year results of the look AHEAD trial. Diabetes Care 2007; 30: 1374–83. 17363746 10.2337/dc07-0048 PMC2665929

[2] Chang S‐H , Stoll CRT , Song J , Varela JE , Eagon CJ , Colditz GA . The effectiveness and risks of bariatric surgery: An updated systematic review and meta‐analysis, 2003–2012. JAMA Surg 2014; 149: 275–87. 24352617 10.1001/jamasurg.2013.3654 PMC3962512

[3] Abu Dayyeh BK , Rajan E , Gostout CJ . Endoscopic sleeve gastroplasty: A potential endoscopic alternative to surgical sleeve gastrectomy for treatment of obesity. Gastrointest Endosc 2013; 78: 530–5. 23711556 10.1016/j.gie.2013.04.197

[4] Fayad L , Adam A , Schweitzer M , *et al*. Endoscopic sleeve gastroplasty versus laparoscopic sleeve gastrectomy: A case‐matched study. Gastrointest Endosc 2019; 89: 782–8. 30148991 10.1016/j.gie.2018.08.030

[5] Sharaiha RZ , Hajifathalian K , Kumar R , *et al*. Five‐year outcomes of endoscopic sleeve gastroplasty for the treatment of obesity. Clin Gastroenterol Hepatol 2021; 19: 1051–7. 33011292 10.1016/j.cgh.2020.09.055

[6] Zhu N , Zhang D , Wang W , *et al*. A novel coronavirus from patients with pneumonia in China, 2019. N Engl J Med 2020; 382: 727–33. 31978945 10.1056/NEJMoa2001017 PMC7092803

[7] Lopez‐Nava G , Asokkumar R . Step‐by‐step approach to endoscopic gastroplasty by a novel single‐channel endoscopic suturing system. VideoGIE 2019; 4: 444–6. 31709325 10.1016/j.vgie.2019.06.008 PMC6831913

[8] Buchwald H , Oien DM . Metabolic/bariatric surgery worldwide 2011. Obes Surg 2013; 23: 427–36. 23338049 10.1007/s11695-012-0864-0

[9] Sone H , Yoshimura Y , Ito H , Ohashi Y , Yamada N . Energy intake and obesity in Japanese patients with type 2 diabetes. Lancet 2004; 363: 248–49. 10.1016/S0140-6736(03)15348-2 14738815

[10] Matsuo T , Ito M , Takata S , Tanaka S , Yoshihara M , Chayama K . Low prevalence of *Helicobacter pylori*‐negative gastric cancer among Japanese. Helicobacter 2011; 16: 415–9. 22059391 10.1111/j.1523-5378.2011.00889.x

